# Activation of S1PR2 on macrophages and the hepatocyte S1PR2/RhoA/ROCK1/MLC2 pathway in vanishing bile duct syndrome

**DOI:** 10.1371/journal.pone.0317568

**Published:** 2025-01-24

**Authors:** Aya Miyagawa-Hayashino, Tetsuya Imura, Toshiaki Takezawa, Maki Hirai, Saya Shibata, Hiroshi Ogi, Takahiro Tsujikawa, Eiichi Konishi

**Affiliations:** 1 Department of Surgical Pathology, Kyoto Prefectural University of Medicine, Kyoto, Japan; 2 Faculty of Pharmacy, Chiba Institute of Science, Chiba, Japan; 3 Department of Pathology and Applied Neurobiology, Kyoto Prefectural University of Medicine, Kyoto, Japan; 4 SCREEN Holdings Co., Ltd., Kyoto, Japan; 5 Department of Otolaryngology–Head and Neck Surgery, Kyoto Prefectural University of Medicine, Kyoto, Japan; Royal Adelaide Hospital, AUSTRALIA

## Abstract

Immunologic bile duct destruction is a pathogenic condition associated with vanishing bile duct syndrome (VBDS) after liver transplantation and hematopoietic stem-cell transplantation. As the bile acid receptor sphingosine 1-phosphate receptor 2 (S1PR2) plays a critical role in recruitment of bone marrow-derived monocytes/macrophages to sites of cholestatic liver injury, S1PR2 expression was examined using cultured macrophages and patient tissues. Bile canaliculi destruction precedes intrahepatic ductopenia; therefore, we focused on hepatocyte S1PR2 and the downstream RhoA/Rho kinase 1 (ROCK1) signaling pathway and bile canaliculi alterations using three-dimensional hepatocyte culture models that form obvious bile canaliculus-like networks. Multiplex immunohistochemistry revealed increased numbers of S1PR2^+^CD45^+^CD68^+^FCN1^+^ inflammatory macrophages and S1PR2^+^CD45^+^CD68^+^MARCO^+^ Kupffer cells in liver tissues showing ductopenia due to graft-versus-host disease and rejection post-liver transplant compared with normal liver. Macrophage expression of proinflammatory cytokines, including MCP1, was reduced following S1PR2 inhibition. Taurocholic acid and S1P2 agonist induced hepatocyte S1PR2 and reduced RhoA/ROCK1 expression, resulting in bile canaliculi dilatation. S1PR2 inhibition reversed the effect on RhoA/ROCK1 expression, resulting in maintenance of bile canaliculi through myosin light chain 2 (MLC2) phosphorylation. Activation of S1PR2 on macrophages and S1PR2 on hepatocytes may disrupt bile canaliculi dynamics in VBDS under regulation by RhoA/ROCK1 through MLC2 phosphorylation.

## Introduction

Vanishing bile duct syndrome (VBDS) is a condition in which chronic cholestasis is associated with progressive loss of intrahepatic bile ducts or ductopenia [1]. VBDS is a heterogenous condition associated with pathologies such as chronic allograft rejection after liver transplantation (LT), chronic graft-versus-host disease (GVHD) after hematopoietic stem cell transplantation (HSCT) [2], and drug-induced liver injury, including Stevens-Johnson syndrome [3]. Persistent exposure to toxic bile salts in addition to direct cholangiocyte damage by drugs or immune-mediated cholangiocyte injury may also cause bile duct loss [1].

Sphingosine 1-phosphate (S1P) functions as a lipid mediator through five different S1P-specific G protein–coupled receptors, S1PR1-S1PR5 [4–6]. S1P receptor 2 (S1PR2) is expressed on the vascular endothelium and vascular smooth muscle of many organs, as well as on leukocytes, such as monocytes, macrophages, and lymphocytes, on the epithelium of some organs, and on neurons [7, 8]. S1PR2 is coupled via G1, Gq, and G12/13-Rho, which inhibits Rac and Akt in the downstream Rho/Rho kinase pathway. S1P2 plays numerous roles, such as preventing the disruption of vascular barrier function, inhibiting vascular formation, maintaining vascular tone, and promoting atherosclerosis in blood vessels, and also in localizing lymphocytes in lymphoid tissues and suppressing lymphoma development [6–8].

In the liver, S1PR2 is one of the receptors upon which bile acids act directly on hepatocytes [9] and cholangiocytes [10, 11]. The S1PR2-mediated signaling pathway can cause liver damage through the regulation of lipid metabolism in non-alcoholic fatty liver disease [12] and through the recruitment and activation of immune cells [13] or activation of stellate cells [14] in cholestatic liver injury. As cholestasis-induced liver injury primarily results from inflammation rather than the toxic effects of bile acids [13, 15], the S1PR2-mediated signaling pathway may link inflammation to cholangiocyte damage in VBDS.

S1PR2 plays a critical role in recruiting bone marrow-derived monocytes/macrophages to sites of cholestatic liver injury [16]. Kupffer cells, resident macrophages in the liver, are derived from circulating monocytes and localize in the liver sinusoids [16]. Excessive influx of bone marrow-derived monocytes/macrophages leads to liver inflammation and fibrosis [17]. Recent research using single-cell RNA sequencing identified two consistently distinct populations of liver resident CD68^+^ macrophages exhibiting unique functional pathways [18–20]. The most conserved gene expression markers in population 1 include *MARCO*, *HMOX1*, and *MRC1*. These genes are optimized for tolerogenic function, suggesting that this population represents immunoregulatory macrophages or Kupffer cells. Population 2 is characterized by enriched expression of genes associated with inflammatory pathways, including *FCN1*, *LYZ*, *S100A8*, and *S100A9*, and thus represents inflammatory macrophages [18–20]. In order to examine the immune characteristics of liver resident macrophages, multiplex immunohistochemistry analysis of the expression of CD45, CD68, S1PR2, MARCO, and FCN1 was performed using human liver tissues showing VBDS [2].

**Fig 1:**
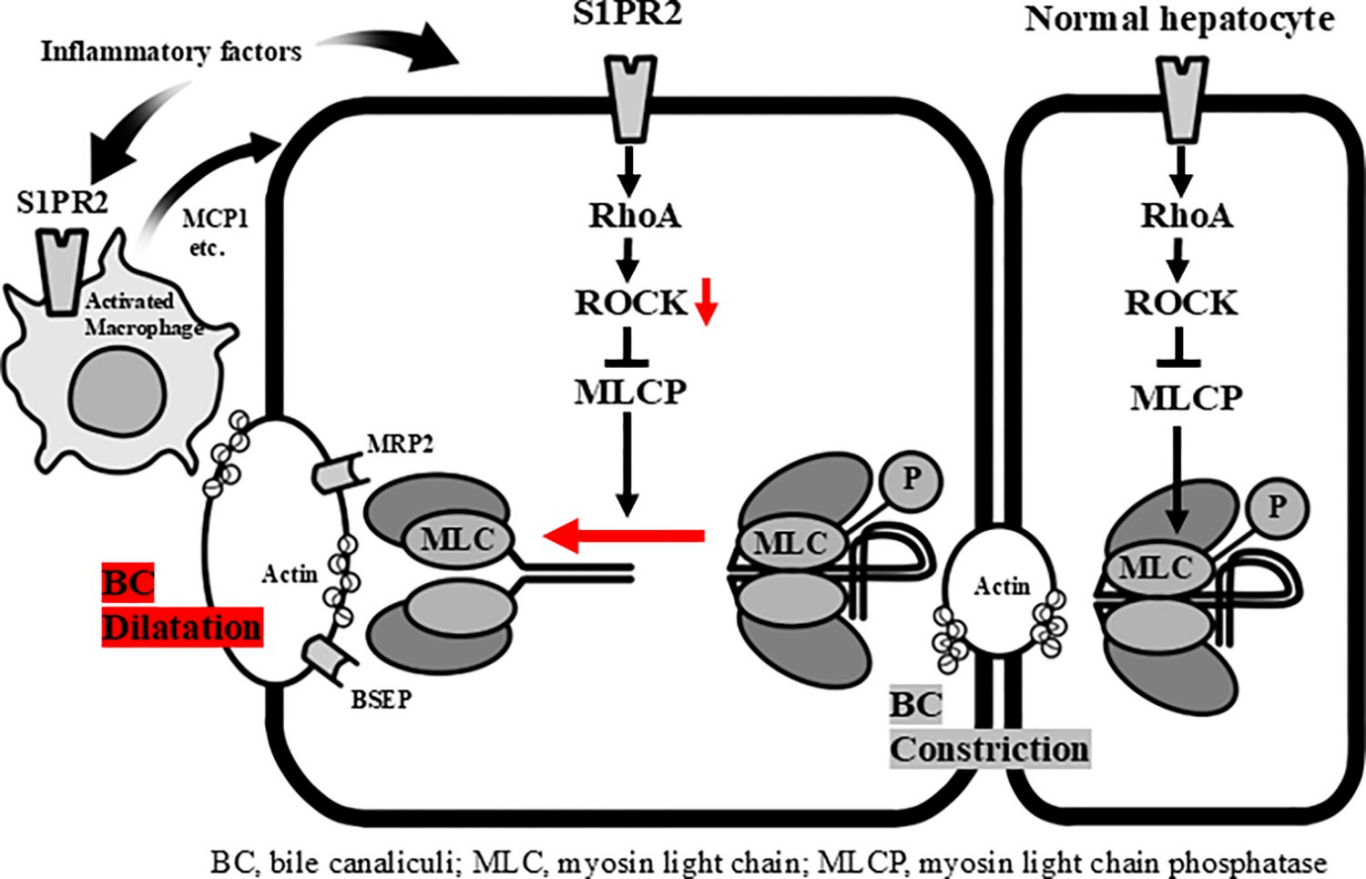
S1PR2 and its downstream molecules, RhoA/ROCK1/myosin light chain 2, in regulating bile canaliculi dynamics. A schematic figure of bile canaliculi (BC) alterations resulting in constriction or dilatation of the canalicular lumen following changes in RhoA/ROCK1/myosin light chain 2 (MLC2) phosphorylation; activation of ROCK1 maintains MLC2 phosphorylation, leading to BC constriction through actin–myosin interaction. Activation of S1PR2 may inhibit ROCK1 activity and cause MLC2 dephosphorylation, leading to BC dilatation.

Bile canaliculi develop from grooves in hepatocytes that form a continuous conduit along the hepatic plates, ultimately connecting to the biliary tree [1]. As we previously reported that destruction of the bile canaliculi in VBDS occurs earlier than morphologic intrahepatic bile duct loss [21], in the present study, we investigated the expression of S1PR2 in the liver, focusing particularly on bile canaliculi and macrophages. We also explored the dynamics of bile canaliculi using three-dimensional oxygenation cell culture models of HepG2 cells from the perspective of S1PR2 signaling and its downstream molecules, the G protein RhoA and Rho kinase 1 (ROCK1) [22], which play an important role in regulating bile canaliculi dynamics [23, 24]. Our results suggest that activation of S1PR2 on both macrophages and hepatocytes may link inflammation to bile canaliculi alterations by inducing bile canaliculi dilatation and destruction through inhibition of RhoA/ROCK1/myosin light chain 2 (MLC2) phosphorylation, leading to the clinical picture of bile duct damage in VBDS (Fig 1).

## Material and methods

### Analysis of publicly available data sets

Single-cell RNA sequencing datasets (https://v22.proteinatlas.org/ENSG00000267534-S1PR2/single+cell+type/liver) retrieved from a previously published study [25] based on healthy human liver tissues were used to analyze single-cell RNA levels of *S1PR2* in each group.

### Patient samples and multiplex immunohistochemistry

**Fig 2:**
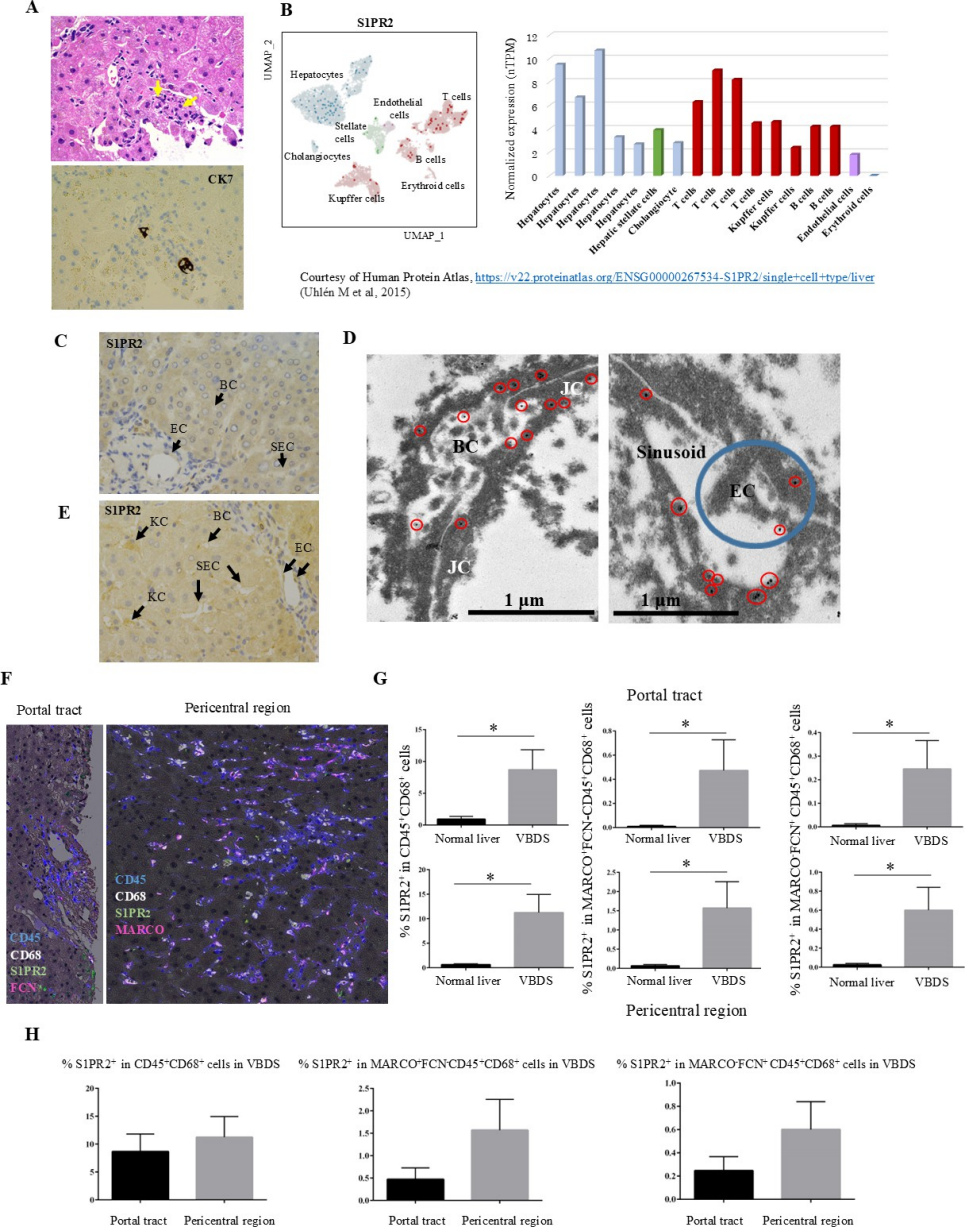
S1PR2 expression in normal liver tissue and vanishing bile duct syndrome (VBDS) and immune characterization of liver macrophages using multiplex immunohistochemistry. (A) Hematoxylin and eosin staining (upper) and CK7 immunohistochemistry analysis (lower) in VBDS associated with graft-versus-host disease (GVHD) after hematopoietic stem cell transplantation. Immunostaining for CK7 highlights atrophy and the paucity of intrahepatic bile ducts (arrows) in the portal tact. (B) Analysis of a single-cell RNA sequencing dataset retrieved from published studies based on healthy human liver tissues revealed that single-cell RNA levels of S1PR2 in hepatocyte clusters were higher than those in other cell type groups. Note the lower RNA expression in Kupffer cells in normal liver compared with hepatocytes. To the left, a UMAP plot illustrates the RNA expression profile of each cell type group. The bar chart to the right shows nTPM levels in each annotated cluster of single cells. Courtesy of Human Protein Atlas, https://v22.proteinatlas.org/ENSG00000267534-S1PR2/single+cell+type/liver (Uhlén et al., 2015). (C) In normal human liver, endothelial cells (ECs) in the portal vein were positive for S1PR2, but no staining was seen in sinusoidal endothelial cells (SEC) or bile canaliculi (BC) by immunohistochemistry (original magnification, ×200). (D) Immunoelectron microscopy revealed the binding sites of S1PR2 around bile canaliculi and sinusoids. BC, bile canaliculi; EC, endothelial cell; JC, junctional complex. Red circles indicate gold particles and blue circles endothelial cells. (E) In a case of GVHD showing VBDS, positive S1PR2 staining was observed in endothelial cells (ECs) in the portal vein and sinusoidal endothelial cells (SEC), bile canaliculi (BC), and Kupffer cells (KC) in the sinusoid, and faint staining was observed in hepatocyte cytoplasm. (F) Left image shows hematoxylin and multiplex staining of S1PR2+ cells in CD45+CD68+FCN1+MARCO− inflammatory macrophages in the portal tract in a case of chronic rejection showing vanishing bile ducts after liver transplantation. Note the paucity of bile ducts. Right image shows hematoxylin and multiplex staining of S1PR2+ cells in CD45+CD68+FCN−MARCO+ Kupffer cells in the pericentral region in a case of antibody-mediated rejection showing vanishing bile ducts after liver transplantation. (G) The percentage S1PR2+ cells among CD45+/CD68+ cells was increased in VBDS compared with normal liver. Liver macrophage populations including CD45+CD68+FCN1+MARCO− inflammatory macrophages and CD45+CD68+FCN1−MARCO+ immunoregulatory macrophages both showed increased S1PR2 expression in the portal tract and pericentral region of lobules in VBDS compared with normal liver. *P<0.05 by unpaired *t*-test. (H) The percentage of S1PR2-expressing macrophages showed an increasing trend in both in FCN-positive and MARCO-positive macrophages in the pericentral region of the liver compared with the portal tracts in VBDS, but the difference was not statistically significant.

Patient samples of tissue from cases of cholestatic liver diseases eventually leading to bile duct atrophy/loss were collected (Fig 2A). Consecutive formalin-fixed paraffin-embedded (FFPE) liver tissues showing GVHD after HSCT (n = 8) or chronic rejection after LT (n = 2), cholangiopathy associated with antibody-mediated rejection for ABO-incompatible LT (n = 15), and normal donor livers taken at LT (n = 20) were acquired at Kyoto Prefectural University of Medicine and Kyoto University Hospital between 2000 and 2022 (S1 Table).

Multiplex immunohistochemistry was performed to evaluate the characteristics of liver macrophages. FFPE tissues were subjected to sequential chromogenic immunostaining with antibodies specific for macrophage lineages, followed by digital scanning using a NonoZoomer S360 (Hamamatsu Photonics, Hamamatsu, Shizuoka, Japan) and subsequent antibody stripping on a single FFPE tissue section, as reported previously [26]. The antibodies and staining conditions used for multiplex immunohistochemistry analyses are shown in S2 Table.

Iteratively digitized images were accurately co-registered using in-house software (SCREEN Holdings Co., Ltd.), which calculates the coordinates of each image relative to a reference image. Using these coordinates, a set of non-compressed TIFF images for each region of interest was extracted. AEC color extraction of single-marker images was performed as previously reported by Ruifrok and Johnston [27]. Single-cell segmentation was performed by an AI system using a WGAN convolutional neural network [28]. Staining intensity was quantified using in-house software (SCREEN Holdings Co., Ltd.). S1PR2 positivity was recorded for 100 cells per image, and the percentage of S1PR2-positive cells over 5 images (total of 500 cells) was calculated for each case of VBDS and for normal liver tissue.

The medical records and archived tissues were accessed for research purposes from January 10, 2023 to January 17, 2024. Authors had access to information that could identify individual participants during the data collection. This retrospective study involving humans was approved by the local Ethics Committee of Kyoto Prefectural University of Medicine [ERB-C-1748-3]. Informed consent was obtained in the form of opt-out on the web-site.

### Immunoelectron microscopy

Immunoelectron microscopy was performed to investigate the subcellular localization of SIPR2 in hepatocytes. Normal human liver was fixed in 4% paraformaldehyde and 0.1% glutaraldehyde in phosphate-buffered saline and embedded in LV White, followed by UV polymerization. The tissue sections were immunolabeled with anti-S1PR2 antibody and secondary antibody coupled to 15-nm colloidal gold and examined under a JEM1230 transmission electron microscope (JEOL, Tokyo, Japan).

### Cell culture and treatment

THP-1 cells (JCRB0112.1) and HepG2-NIAS cells (RCB4679) were provided by the RIKEN BioResource Center (Tsukuba, Ibaraki, Japan). THP-1 human monocytes were maintained in RPMI-1640 medium containing 10% heat-inactivated fetal bovine serum (Sigma-Aldrich, St. Louis, MO, USA) and 1% penicillin-streptomycin and incubated in a 5% CO_2_ incubator at 37°C. THP-1 cells were stimulated with 100 ng/mL phorbol 12-myristate 13-acetate (PMA; Sigma-Aldrich) for 24 h to induce differentiation into macrophages, unless otherwise indicated [29].

HepG2-NIAS cells were maintained in Dulbecco’s modified Eagle medium, low glucose, pyruvate (Thermo Fisher Scientific, Waltham, MA, USA), supplemented with 10% heat-inactivated fetal bovine serum (Sigma-Aldrich), 20 mM HEPES (Thermo), 100 units/mL penicillin, and 100 μg/mL streptomycin (Thermo) and incubated in a 5% CO_2_ incubator at 37°C.

For drug experiments, cells were treated with 100 μM TCA (Sigma Aldrich), 100 nM S1P2 agonist (CYM5520, Cayman, Ann Arbor, MI, USA), or 50 μM CyA (Tokyo Chemical, Tokyo, Japan) for 24 h; 10 μM ROCK inhibitor Y27632 dihydrochloride (Med Chem Express, Monmouth Junction, NJ, USA) for 1 h; or 10 μM selective S1P2 antagonist (JTE-013, Selleck, Houston, TX, USA) for 1 h.

For RNA interference assays, THP-1 cells were resuspended in 100 nM siRNA targeting *S1PR2* (MBS8235059, MyBioSourse, San Diego, CA) or scrambled negative control siRNA using TransIT-TKO Transfection Reagent (TakaraBio, Shiga, Japan) and incubated for 48 h.

### Two-step culture of HepG2-NIAS cells

No culture strains suitable for studying the structure of bile canaliculi are available. HepG2 cells exhibit poor hepatic function, with almost no CYP3A4 activity and poor formation of bile canaliculus–like networks when cultured in monolayers on plastic plates [30]. One of the coauthors (Takezawa) established three-dimensional oxygenation cell culture models of HepG2-NIAS cells using collagen vitrigel membranes (CVMs) (Kanto Chemical, Tokyo, Japan), which form obvious bile canaliculus–like networks and exhibit rapid activation of liver-specific functions [30]. HepG2-NIAS cells are cultured using a two-step method with CVM chambers; the first step is liquid-solid interface culture for 2 days, followed by the second step, liquid-gas phase culture for 1 day [30].

### Quantitative real-time PCR (qPCR)

Total RNA was extracted from THP-1 or HepG2-NIAS cells using NucleoSpin RNA (Takara). cDNAs were synthesized using SuperScript IV VILO Master Mix (Thermo). qPCR analysis of *S1PR2*, *MCP1*, *TNFα*, *IL6*, *RHOA*, and *ROCK1* expression was performed using FastStart Universal Sybr Rox (Roche, Basel, Switzerland) on a StepOne Plus system (Thermo) with *Gapdh* as an endogenous control (S3 Table).

### Flow cytometry

THP-1 cells were treated with PMA for 24 h and S1P2 agonist for an additional 24 h with or without pretreatment with JTE-013 and then blocked with human Fc-receptor blocking reagent (MBL, Tokyo, Japan), followed by incubation with CoraLite Plus 488-conjugated anti-S1PR2 (CL488-21180, Proteintech, Rosemont, IL) and isotype control antibodies (CL488-30000, Proteintech). The cells were then analyzed using a FACSCelesta multicolor flow cytometer (BD Biosciences, Franklin Lakes, NJ, USA).

### Immunofluorescence analysis

HepG2-NIAS cells cultured on a CVM chamber were fixed with 4% paraformaldehyde phosphate buffer. Immunofluorescence staining was performed with F-actin (PHDH1, Acti-stain 555 phalloidin, CYT, Denver, CO, USA) and antibodies against BSEP (sc-74599, Clone F-6, Santa Cruz Biotechnology, Dallas, TX, USA) and MRP2 (ab3373, Clone M2III-6, Abcam) and detected using a TSA Plus Fluorescence kit (PerkinElmer, Inc., Waltham, MA, USA). Nuclei were stained with DAPI (Dojindo, Kumamoto, Japan) and examined under an LSM900 confocal microscope (Carl Zeiss, Jena, Germany).

### Quantitative analysis of bile canaliculi area

The area of bile canaliculi was determined from the outer diameter using NIS-Elements imaging software, ver. 5.01 (Nikon Corp., Tokyo, Japan). The mean area of bile canaliculi for 5 images of F-actin staining of HepG2-NIAS cells on a CVM chamber was determined.

### Protein analysis

HepG2-NIAS cells were lysed for 1 h on ice in RIPA buffer (Nakalai Tesque, Kyoto, Japan) containing protease inhibitor (Cell Signaling, Danvers, MA, USA). Automated Western blotting was performed using a Jess instrument (ProteinSimple, Bio-Techne, Minneapolis, MN, USA) following the manufacturer’s instructions. Antibodies against RhoA (sc-418, Clone 26C4, Santa Cruz), ROCK1 (#4035, Clone C8F7, Cell Signaling), S1PR2 (Proteintech), and Gapdh (ab9485, Abcam) were used for the analysis.

### Enzyme-linked immunosorbent assay (ELISA)

THP-1 cells were stimulated to differentiate into macrophages by treatment with PMA for 24 h, after which S1PR2 siRNA was added and the cells incubated for an additional 24 h and then treated with S1P2 agonist or activated with 10 μg/mL lipopolysaccharide (LPS; Wako Chemicals, Osaka, Japan) for an additional 24 h. Levels of GM-CSF, IFNγ, IL4, IL6, IL10, IL12, MCP1, and TNFα in the culture medium were determined using a human M1/M2/MDSC cytokine multiplex ELISA kit (Arigobio, Hsinchu City, Taiwan).

HepG2-NIAS cell lysates treated with 50 μM CyA with or without JTE-013 or Y27632 pretreatment were collected, and phosphorylated MLC2 was quantified using a human phosphorylated MLC2 ELISA kit (MyBioSource, San Diego, CA, USA). Absorbance for ELISAs was measured at 450 nm using GloMax Discover, ver. 3 (Promega, Madison, WI, USA).

### Statistical analysis

Statistical analyses were performed using GraphPad Prism 6 (MDF Co., Ltd., Tokyo, Japan). Differences were assessed using the unpaired Student’s *t*-test. Differences between groups were analyzed using one-way analysis of variance with the Tukey or Dunnett test. Data are presented as the mean with standard error of the mean (SEM). Statistical significance was indicated at *P*<0.05.

## Results

### S1PR2 expression in normal liver tissue and VBDS after transplantation

Analysis of single-cell RNA sequencing datasets retrieved from published studies based on healthy human liver tissues revealed that single-cell RNA levels of *S1PR2* in hepatocyte clusters were higher than those in other types of cells. The RNA expression levels in Kupffer cells were lower than those in hepatocytes in normal liver (Fig 2B).

Immunohistochemistry analysis of normal human liver tissue revealed weak positivity for S1PR2 in the cytoplasm of hepatocytes and endothelial cells in the portal tracts and central veins (Fig 2C), but no expression was observed in Kupffer cells, sinusoids, or bile canaliculi. As the specific localization of S1PR2 in hepatocytes could not be clearly determined by light microscopy, we examined the subcellular binding sites of S1PR2 in hepatocytes in normal human liver tissue using immunoelectron microscopy. Binding sites of S1PR2 in hepatocytes were detected primarily around bile canaliculi (pericanalicular cytoplasm) and sinusoids (Fig 2D). Thus, in normal liver, the expression levels of S1PR2 on bile canaliculi, sinusoids, and Kupffer cells were below the detection sensitivity of immunohistochemistry (Fig 2C). In VBDS after LT or HSCT, S1PR2 positivity was observed on endothelial cells in the portal veins and sinusoids, bile canaliculi, Kupffer cells in sinusoids, infiltrating macrophages in portal tracts, and the cytoplasm of hepatocytes (Fig 2E).

### High S1PR2 expression in liver macrophage populations in VBDS by multiplex immunohistochemistry analysis

Immunologic characteristics of liver macrophages were evaluated using multiplex immunohistochemistry (Fig 2F). The percentage of S1PR2^+^ cells was higher among CD45^+^CD68^+^ macrophages in the portal tract and pericentral region in liver with VBDS compared with normal liver. S1PR2 expression was increased in CD45^+^CD68^+^FCN1^+^MARCO^−^ inflammatory macrophages and CD45^+^CD68^+^FCN^−^MARCO^+^ immunoregulatory macrophages both in the portal tract and pericentral region in VBDS liver compared with normal liver (Fig 2G). In VBDS specimens, the percentage of S1PR2-expressing macrophages tended to be higher in both FCN-positive and MARCO-positive macrophages in the pericentral region compared with S1PR2-expressing macrophages in the portal tracts, although the difference was not statistically significant (Fig 2H). This result may reflect the predominantly pericentral localization of bilirubin deposition in hepatocytes and phagocytosis of bile by Kupffer cells in the sinusoidal spaces [1].

### S1RR2 mediates macrophage activation leading to proinflammatory cytokine production

**Fig 3:**
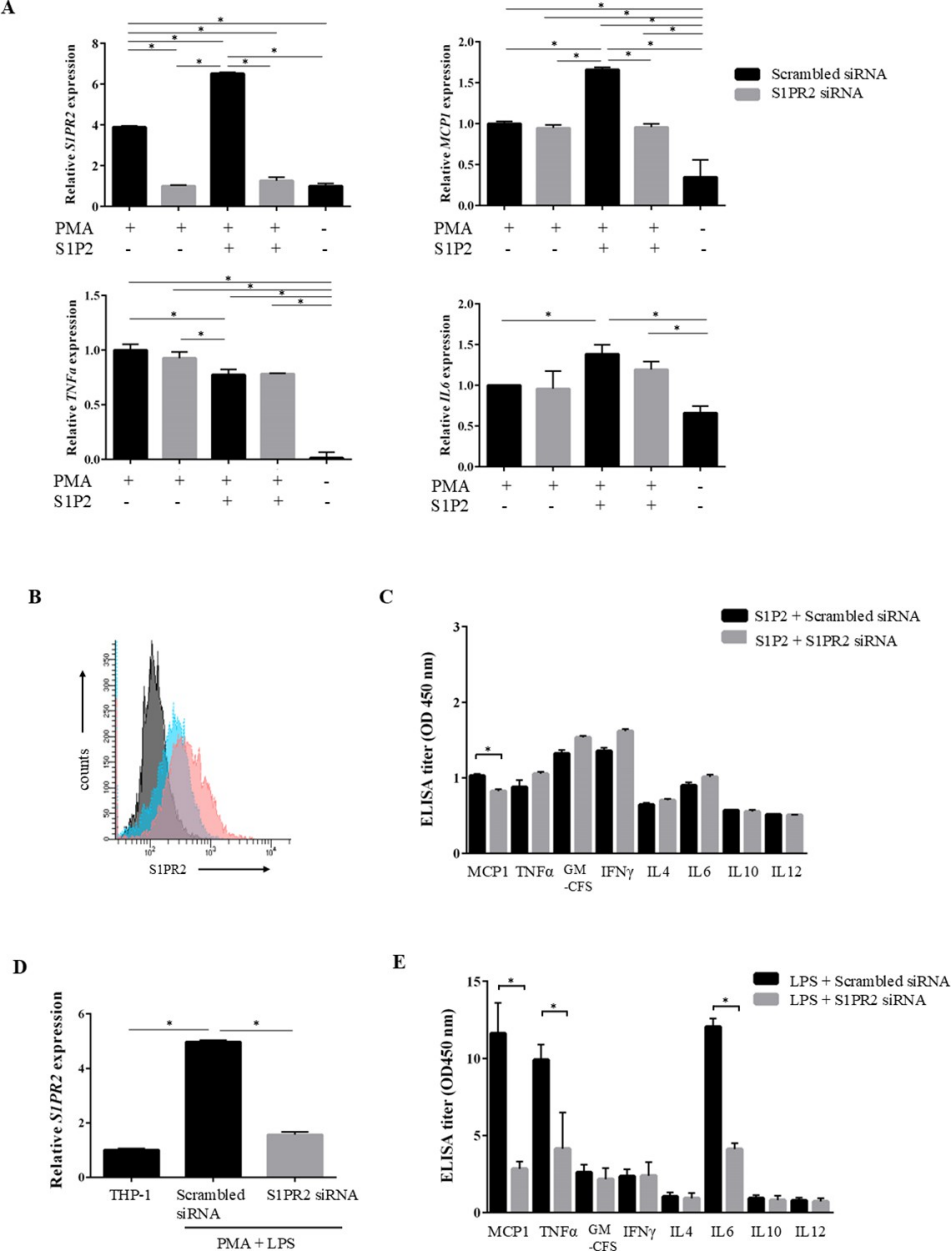
S1PR2 inhibition reduces the levels of proinflammatory cytokines, including MCP1 in LPS- and S1P2-stimulated macrophages. (A) THP-1 cells were induced to differentiate into macrophages by PMA treatment and showed increased expression of *S1PR2*. Treatment with an S1P2 agonist (CYM5520) upregulated mRNA expression levels of *S1PR2* and *MCP1* in macrophages, and *S1PR2* knockdown significantly inhibited the expression of *S1PR2* and *MCP1* compared with macrophages treated with scrambled siRNA. (B) Macrophages were analyzed by flow cytometry for the expression of S1PR2 with S1P2 agonist. Representative histogram of S1PR2 expression with S1P2 agonist (pink line); JTE-013 inhibited expression (blue line). Black line represents the isotype control. One representative result of three is shown. (C) ELISA showing that supernatant levels of S1P2-induced MCP1 were significantly reduced by *S1PR2* knockdown in macrophages. (D) The expression of *S1PR2* mRNA was significantly elevated on PMA-induced differentiated macrophages stimulated with LPS. *S1PR2* siRNA treatment effectively silenced the corresponding target gene by 70%, as assessed by qPCR. (E) ELISA showing that supernatant levels of proinflammatory cytokines, including MCP1, TNF-α, and IL-6, were increased in macrophages stimulated with LPS. *S1PR2* knockdown significantly blocked the production of these cytokines by macrophages. *P<0.05 by one-way ANOVA, Tukey test in (A),*P<0.05 by unpaired *t*-test in (C-E). Results are the mean ± SEM of triplicate experiments.

We also examined the effect of S1P2 agonist treatment on proinflammatory cytokine production by macrophages. Treatment with S1P2 agonist upregulated mRNA expression of *S1PR2* and *MCP1* in macrophages, and *S1PR2* knockdown significantly inhibited this upregulation compared with macrophages treated with scrambled siRNA. No differences in transcript levels of *TNFα* and *IL6* were observed between control macrophages and S1P2-treated macrophages with *S1PR2* knockdown (Fig 3A). Flow cytometric analysis of a single-cell suspension of S1P2-activated differentiated macrophages showed an increase in surface S1PR2 expression, and treatment with the selective S1PR2 antagonist JTE-013 suppressed S1PR2 expression (Fig 3B). ELISA results showed no significant differences in the supernatant levels of proinflammatory cytokines, except MCP1, between macrophages with *S1PR2* knockdown and those treated with scrambled siRNA when S1P2 agonist was applied (Fig 3C).

As cytokine release was thought to be too low to observe a significant difference when cells were treated with S1P2 agonist, cytokine induction via S1PR2 in response to LPS stimulation was investigated. Expression of S1PR2 on THP-1 cells was induced by PMA and LPS stimulation and downregulated by treatment with *S1PR2* siRNA, which was confirmed by qPCR (Fig 3D). Supernatant levels of various proinflammatory cytokines, including MCP1, TNFα, and IL6, were increased, and *S1PR2* knockdown significantly blocked the production of these cytokines by LPS-activated macrophages. Levels of anti-inflammatory cytokines such as IL4 and IL10 were unchanged following *S1PR2* knockdown (Fig 3E).

Although S1P2 treatment slightly increased S1PR2 expression and promoted *MCP1* transcription and MCP1 secretion via S1PR2 in macrophages, treatment with S1P2 alone exhibited a weaker cytokine-inducing effect. Increased S1PR2 expression was positively correlated with secretion of the proinflammatory cytokines MCP1, TNFα, and IL6 following LPS stimulation. The findings suggest that S1PR2 signaling enhances proinflammatory cytokine production in response to LPS-mediated innate signaling in activated macrophages [31].

### TCA and S1P2 agonist induced the dilatation of bile canaliculi, and S1PR2 inhibition reversed the change in bile canaliculi size in three-dimensional oxygenation cell culture models

**Fig 4:**
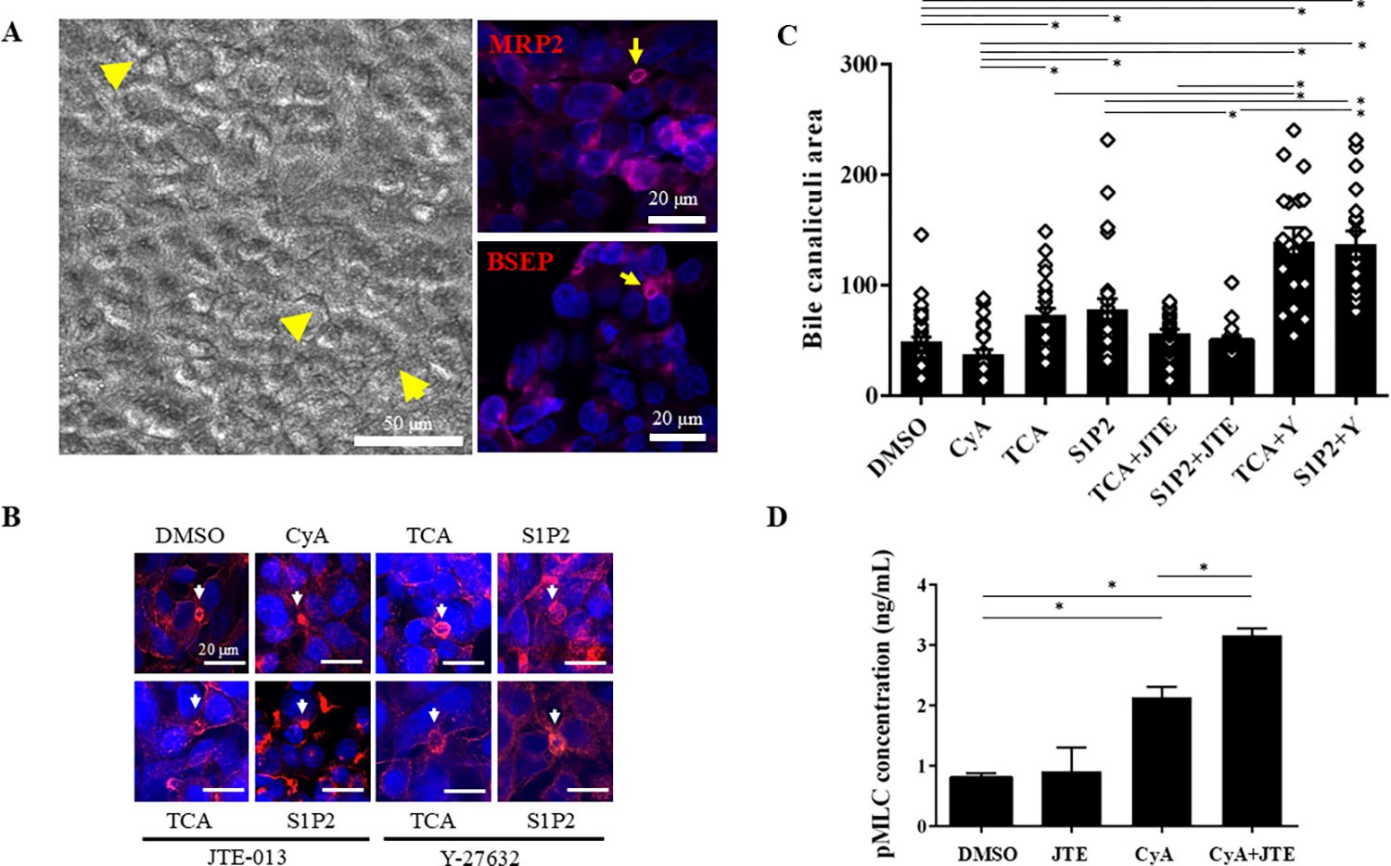
Treatment with TCA and S1P2 agonist leads to bile canaliculi dilatation, and inhibition of S1PR2 expression reverses the change in the size of the bile canaliculi lumen; inhibition of S1PR2 expression increases MLC2 phosphorylation. (A) Well-formed bile canaliculus–like networks generated using a novel culture method with collagen vitrigel membrane (CVM) chambers (arrowheads). Canalicular transporters, multidrug resistance–related protein 2 (MRP2/ABCC2), and bile salt export pump (BSEP/ABC11) are distributed on the canalicular membranes (arrows). (B) Effects of TCA and S1P2 on bile canaliculi with or without inhibitors (JTE-013 or Y27632). Bile canaliculi are highlighted red by pericanalicular F-actin (arrows). For comparison, bile canaliculi constriction resulting from treatment with cyclosporine A (CyA) (50 μM) for 24 h is shown. TCA and S1P2 exposure led to bile canaliculi dilatation, in contrast to the control. JTE-013 negated the effect of TCA- or S1P2-induced dilatation of the bile canaliculi lumen. In the presence of Y27632, TCA or S1P2 induced strong dilatation of bile canaliculi. (C) Quantification of the bile canaliculi area with various compounds tested using CVM chambers. Exposure to CyA resulted in constriction of the bile canaliculi lumen, and treatment with TCA or S1P2 resulted in dilatation of the bile canaliculi lumen. TCA exposure following pretreatment with the selective S1PR2 antagonist JTE-013 for 1 h reduced bile canaliculi dilatation. S1P2 exposure following pretreatment with JTE-013 for 1 h restored the normal bile canaliculi area. The bile canaliculi lumen was significantly dilated with TCA or S1P2 exposure following pretreatment with Y27632. (D) ELISA showed that MLC2 phosphorylation increased following treatment with the constrictor, CyA, and inhibition of S1PR2 increased the level of phosphorylated MLC2 under the condition of CyA treatment. *P<0.05 by one-way ANOVA, Tukey’s test.

Given the increased S1PR2 expression and S1PR2-influenced proinflammatory cytokine secretion by activated macrophages, we investigated bile canaliculi dynamics relative to S1PR2 in HepG2-NIAS cells. Experiments using a novel two-step culture method with CVM chambers [30, 32] revealed that bile canaliculus-like networks were well formed at day 2. The canalicular transporters multidrug-resistance-related protein 2 (MRP2/ABCC2) and bile salt export pump (BSEP/ABC11) were distributed on the canalicular membranes, as reported previously [32] (Fig 4A). As treatment with TCA is known to elongate the bile canaliculi network [33], and drug-induced cholestasis is strongly associated with deformation of the bile canaliculi [23], we investigated bile canaliculi dynamics via S1PR2 in HepG2-NIAS cells using TCA, both predominant BSEP substrate and S1PR2 ligand [24], and S1P2 agonist. CyA (which acts as a ROCK activator and constrictor of bile canaliculi) and Y27632 (a ROCK inhibitor and a dilater of bile canaliculi) were used for comparison [23, 24]. CyA is widely used as an immunosuppressant after LT and HSCT [34].

Bile canaliculi structures were well recognized by the pericanalicular distribution of F-actin (Fig 4B). The area of bile canaliculi was evaluated after short exposures to various drugs. CyA exposure resulted in constriction of the bile canaliculi. The mean bile canaliculi area was 37.31±4.02 (SE) μm^2^ in cells exposed to CyA for 24h as compared with 48.99±4.01 μm^2^ in the control (DMSO). Treatment with TCA for 8h (73.07±5.83 μm^2^) or S1P2 for 24h (78.15±9.29 μm^2^) resulted in dilatation of the bile canaliculi. Cells treated with TCA for 8h following pretreatment with JTE-013 exhibited reduced bile canaliculi dilatation (56.38±3.79 μm^2^). Treatment with S1P2 for 24h in cells pretreated with JTE-013 resulted in a return to normal bile canaliculi area (50.41±3.81 μm^2^). TCA and S1P2 administration after a 1-h treatment with Y27632 resulted in a further expansion of the bile canaliculi area (TCA+Y 139.7±12.15 μm^2^; S1P2+Y 137.2±11.94 μm^2^). The difference in bile canaliculi area was statistically significant as compared with the control or that with CyA treatment, except for cells treated with TCA or S1P2 after pretreatment with JTE-013 (*P*<0.05) (Fig 4C).

### ROCK1 may maintain the structure of bile canaliculi via phosphorylation of MLC2

The involvement of phosphorylated MLC2 in the constriction or dilatation of bile canaliculi was examined by measuring phosphorylated MLC2 in HepG2-NIAS cells by ELISA. CyA, a ROCK activator that causes progressive constriction of bile canaliculi via phosphorylation of MLC2, was used for this analysis [23, 24]. MLC2 phosphorylation increased with CyA concentration, and inhibition of S1PR2 by JTE-013 increased the level of phosphorylated MLC2 under the condition of CyA treatment (Fig 4D).

### TCA and S1P2 agonist induced the expression of S1PR2 and reduced RhoA/ROCK1 expression in hepatocytes

**Fig 5:**
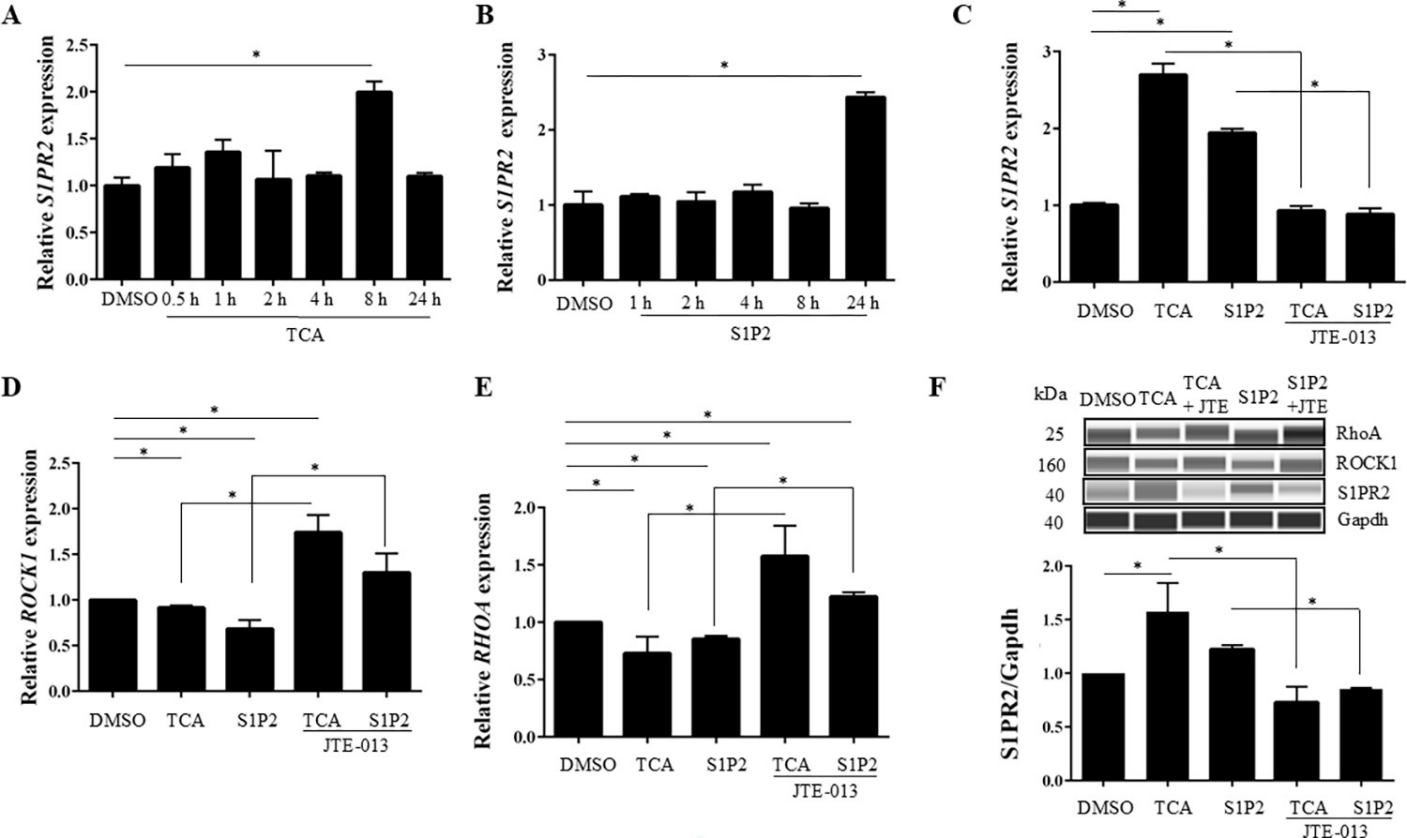
Treatment with TCA and S1P2 agonist induces S1PR2 expression and attenuates ROCK1/RHOA expression; inhibition of S1PR2 reverses TCA- or S1P2-mediated effect on ROCK1/RHOA expression in HepG2-NIAS cells. (A) Induction of *S1PR2* mRNA expression by TCA treatment was transient, reaching a maximum at 8 h and returning to the basal level 24 h after treatment in HepG2-NIAS cells. (B) Induction of *S1PR2* mRNA expression following 24-h treatment of HepG2-NIAS cells with S1P2 agonist. (C) *S1PR2* mRNA expression was significantly increased with TCA or S1P2 agonist stimulation as compared with the control and significantly reduced after pretreatment with JTE-013, an antagonist of S1PR2. (D) TCA or S1P2 agonist administration attenuated *ROCK1* mRNA expression. Pretreatment with JTE-013 reversed the TCA- or S1P2-mediated decrease in *ROCK1* expression. (E) *RHOA* mRNA expression exhibited a tendency similar to that of *ROCK1*. (F) Western blotting analysis of RhoA, ROCK1, and S1PR2 expression in HepG2-NIAS cells treated with TCA or S1P2 agonist with or without pretreatment with JTE-013. TCA- or S1P2-mediated S1PR2 expression was suppressed following pretreatment with JTE-013. The changes in protein levels of RhoA and ROCK1 were not significant. *P<0.05 by one-way ANOVA, Dunnett’s multiple comparison test compared with DMSO control in (A) and (B). One-way ANOVA, Tukey’s test in (C), (D), and (E). Results are the mean ± SEM of triplicate experiments.

As RhoA/ROCK1 are known downstream molecules in the S1PR2 signaling pathway [22] and regulates bile canaliculi dynamics [23, 24], RhoA/ROCK1 expression of S1PR2 ligand in response to TCA and S1P2 agonist was examined in HepG2-NIAS cells. S1PR2 mRNA increased significantly upon treatment with 100 μM TCA for 8 h and 100 nM S1P2 agonist for 24 h compared with the control (Fig 5A and 5B). Thus, a subsequent experiment was performed with 8 h exposure to TCA and 24 h exposure to S1P2 agonist in HepG2-NIAS cells. JTE-013 restored the TCA- and S1P2-induced increase in S1PR2 mRNA expression (Fig 5C). Decreases in ROCK1 and RhoA mRNA expression were observed in response to treatment with TCA and S1P2 agonist (Fig 5D and 5E). Pretreatment with JTE-013 reversed the TCA- and S1P2-mediated changes in ROCK1 and RhoA mRNA expression. Immunoblotting confirmed the results of S1PR2 transcript level analyses, but the changes in protein levels of RhoA and ROCK1 were not significant (Fig 5F).

Collectively, these data show that TCA and S1P2 agonist induce the expression of S1PR2 and reduce the expression of *RhoA/ROCK1* mRNA in HepG2-NIAS cells, resulting in dilatation of bile canaliculi. S1PR2 inhibition increased MLC2 phosphorylation under CyA exposure and returned the area of the bile canaliculi to the normal size. These results suggest that S1PR2 negatively regulates the downstream RhoA/ROCK1 pathway, which maintains the structure of bile canaliculi through MLC2 phosphorylation.

## Discussion

We previously reported that the destruction of bile canaliculi in VBDS occurs earlier than morphological intrahepatic bile duct loss [21]. In this study, we investigated S1PR2 expression in VBDS and the role of the S1PR2/RhoA/ROCK1 pathway in bile canaliculi changes induced by exposure to TCA and S1P2 agonist in HepG2-NIAS cells. S1PR2 signaling promotes macrophage activation [22] and may exert pro-inflammatory activity [6, 35, 36]. In mouse models of atherosclerosis, S1PR2 signaling in activated macrophages was shown to promote production of proinflammatory cytokines (e.g., IL-1β, IL-18), and S1PR2 may retain macrophages in atherosclerotic plaques [36]. The other study showed that knockout of S1PR2 reduces bronchial leakage in murine LPS-induced lung injury [35]. In this study, we demonstrated that macrophages in liver tissues in VBDS express high levels of S1PR2. S1PR2-expressing macrophages were distributed in the pericentral areas as well as in the portal tracts and increased S1PR2 expression was seen both in MARCO^+^ immunoregulatory macrophages and FCN^+^ inflammatory macrophages in VBDS compared with normal liver. Furthermore, *S1PR2* knockdown in macrophages suppressed proinflammatory cytokine secretion. The findings suggest that S1PR2 signaling promotes macrophage activation and the immune response [22].

S1PR2 appears to regulate cytoskeletal organization [6, 37] and control the vascular endothelial barrier function [22]. We therefore hypothesized that S1PR2 affects the bile canaliculi structure and in turn bile leakage. Under cholestatic conditions in VBDS, it is possible that the lipid constituents of bile are taken up by hepatocytes and stimulate S1PR2 expression on the sinusoidal membrane. Destruction of the bile duct epithelium by immune cells results in impaired bile flow, which can induce S1PR2 expression on the canalicular membrane; or, S1PR2 may diffuse across the hepatocytes from the sinusoidal membrane to the canalicular membrane. Our study found that activation of S1PR2 inhibited the downstream RhoA/ROCK1 signaling pathway, which normally plays an important role in maintaining the structure of bile canaliculi, resulting in destruction of the bile canaliculi. Inhibition of S1PR2 expression reversed the effect on ROCK1 expression in HepG2-NIAS cells treated with TCA or S1P2 and thereby maintained the normal size of the bile canaliculi. Via S1PR2, TCA and S1P2 may participate in hepatocytes in the disruption of bile canaliculi dynamics normally regulated by RhoA/ROCK1, and this effect probably occurs through phosphorylation of the regulatory light chain of the cytoskeletal protein non-muscle myosin II [38, 39]. Changes in the phosphorylation state of MLC2 are known to affect the contraction and dilatation of bile canaliculi [23, 24].

Previous studies in a murine model of DDC-induced cholestatic liver fibrosis found that S1PR2 plays a role in the migration of bone marrow-derived monocytes/macrophages to sites of cholestatic liver injury [16] and that inhibition of S1PR2 expression attenuates liver injury and fibrogenesis [14]. S1PR2 signaling suppresses PIK3 signaling, resulting in a dampening of pro-survival Akt activity in germinal center B-cells, and S1PR2 could thus exert a marked effect on control of Akt activation downstream of innate signals such as TLR ligands [7]. Our study showed that S1PR2 signaling has a significant effect on the control of proinflammatory cytokine secretion in macrophages in response to stimulation with LPS, a known TLR ligand. S1P2 stimulation alone may only weakly induce proinflammatory cytokine expression. However, S1P2 may exert a synergistic effect when another stimulus (e.g., the TLR-mediated signaling pathway) is introduced. S1P2 exposure may have a priming effect, leading to S1PR2 overexpression and enhance cytokine induction via LPS. It is possible that the effect of S1PR2-expressing activated macrophages on hepatocytes and the S1PR2-mediated signaling pathway may link inflammation to bile canaliculi damage through inhibition of RhoA/ROCK1/MLC2 phosphorylation, leading to the clinical picture of bile duct damage in VBDS. This scenario is in agreement with recent reports indicating that S1PR2 exerts a damaging effect on intestinal vascular endothelial barrier function and macrophages in inflammatory bowel disease [22]. Exploring bile canaliculi dynamics using three-dimensional oxygenation cell culture models of hepatocytes from the viewpoint of S1PR2 signaling through S1PR2 expression on macrophages could help elucidate the pathogenesis of VBDS.

Targeting S1PR2 might therefore be a potential therapeutic strategy, however, the S1PR2-mediated signaling pathway in the regulation of cell proliferation and cancer progression remains controversial [6, 40]. TCA-induced increased expression of S1PR2 and cancer cell migration have been detected in cholangiocarcinoma or esophageal adenocarcinoma [10, 41], while, S1PR2-mediated signaling are reported to be associated with anti-cancer signaling in various cancers, including hepatocellular carcinoma, breast cancer or myeloma [42–45] and some hormones and anti-cancer agents were found to activate the expression of S1PR2 [42]. More detailed experimental work is needed to identify the exact role of S1PR2 in the regulation of cell proliferation and cancer progression.

In conclusion, our study suggested that S1PR2-mediated signaling between macrophages and hepatocytes may disrupt bile canaliculi dynamics in VBDS, regulated by RhoA/ROCK1 through MLC2 phosphorylation. To enhance clinical outcomes in VBDS, future research should focus on developing targeted therapies that modulate S1PR2 signaling [6, 14]. This could involve the design of selective S1PR2 inhibitors that minimize off-target effects while preserving bile canaliculi integrity and preventing ductopenia. Additionally, exploring combination therapies that simultaneously target S1PR2 and related inflammatory pathways may offer synergistic benefits. Further studies are needed to validate these findings in larger cohorts and animal models, and to investigate the potential for early biomarker identification of patients at risk for VBDS. Ultimately, integrating these strategies into clinical practice could improve long-term outcomes for patients undergoing LT and HSCT.

## Supporting information

S1 Table Patient demographics. (XLSX)

S2 Table List of antibodies and staining conditions for multiplex immunohistochemistry. (XLSX)

S3 Table Primer sequences for quantitative real time PCR. (DOCX)
