# Supplementary material for: Activation of S1PR2 on macrophages and the hepatocyte S1PR2/RhoA/ROCK1/MLC2 pathway in vanishing bile duct syndrome
Source: PLoS One. 2025 Jan 24;20(1):e0317568. doi: 10.1371/journal.pone.0317568 (PMC11760576; doi:10.1371/journal.pone.0317568)
Supplement: S3 Table — (DOCX) [file pone.0317568.s003.docx]

S3 Table. Primer sequences for quantitative real time PCR

*S1PR2*: 5’-TGGAAACGCAGGAGACGACCTC-3’

and 5’-CGAGTGGAACTTGCTGTTTCGG-3’

*MCP1*: 5′-AGAATCACCAGCAGCAAGTGTCC-3′

and 5′-TCCTGAACCCACTTCTGCTTGG-3′

*TNFα*: 5’-CTCTTCTGCCTGCTGCACTTTG -3’

and 5’-ATGGGCTACAGGCTTGTCACTC-3’

*IL6*: 5’-AGACAGCCACTCACCTCTTCAG-3’

and 5’-TTCTGCCAGTGCCTCTTTGCTG-3’

*RHOA*: 5’-TCTGTCCCAACGTGCCCATCAT-3’

and 5’-CTGCCTTCTTCAGGTTTCACCG-3’

*ROCK1*: 5’-GAAACAGTGTTCCATGCTAGACG-3’

and 5’-GCCGCTTATTTGATTCCTGCTCC-3’.

*Gapdh*: 5′-AACTTTCGATGGTAGTCGCCG-3′

and 5′-CCTTGGATGTGGTAGCCGTTT-3′.
